# “Moving Freely in Space with Power and Not Be Afraid”: An Interpretative Phenomenological Analysis of the Experiences of Elite Rowers with Visual Impairment

**DOI:** 10.3390/ijerph192114059

**Published:** 2022-10-28

**Authors:** Jason Rich, Lauren J. Lieberman, Pamela Beach, Melanie Perreault

**Affiliations:** 1 Wegmans School of Health and Nutrition, Rochester Institute of Technology, Rochester, NY 14623, USA; 2 Kinesiology, Sport Studies, & Physical Education, SUNY Brockport, Brockport, NY 14420, USA

**Keywords:** accessibility, Paralympics, stigma

## Abstract

Due to the accessibility of the sport of rowing for individuals with visual impairment (VI), rowers with VI are a unique population because they have the potential to be just as competitive outside of Para-rowing as sighted rowers. The purpose of this study was to explore the lived experiences of elite rowers with VI in relation to the benefits and challenges of those experiences as well as their peer and coach relationships. Adopting an Interpretative Phenomenological Analysis (IPA) approach to guide data collection, analysis, and interpretation, eight participants with VI who rowed on the national level or higher were recruited and interviewed. The analysis identified four major themes: Empowerment Through Rowing, Rowing Through Feel, Changing Perceptions, and Forming Influential Relationships. The identified themes illustrate the influence of rowing upon the participants’ lives, careers, and successes, as well as the disability awareness of the coaches and peers influencing their experiences.

## 1. Introduction

Rowing is a sport with a long history and tradition, dating back several millennia as well as three centuries as an organized sport [1]. What started as competition between watermen racing on the Thames for a cash prize has turned into an Olympic staple with crews from dozens of countries racing in 14 different boat classes. Boats are comprised of one, two, four, or eight rowers sitting on sliding seats with their feet strapped into foot plates facing the back of the boat [2]. Four- or eight-person boats are steered in the back of the boat by a coxswain (i.e., a shorter and lighter non-rowing teammate whose entire role is to steer and provide verbal commands to the rowers). Smaller coxless boats are steered by the rower sitting closest to the front of the boat by verbally calling for pressure on one side of the boat or using steering attached to a foot.

The World Rowing Federation (FISA) is the world governing body for rowing. FISA introduced Para-rowing, originally adaptive rowing, in the rowing world championships in 2002, and it premiered in the Paralympics (international multisport event that is supported by and runs immediately following the Olympics for athletes with physical, visual, and some intellectual impairments) in 2008 [3,4]. Para-rowing has numerous boat classes based on the rower’s classification and level of physical ability. While many of the classifications are related to physical disability, four Para-rowing events (mixed men’s and women’s coxed four, mixed men’s and women’s double, men’s pair, and women’s pair) also allow for rowers with visual impairment (VI). Whether a two-person boat or a four-person boat, no more than half the athletes’ impairments can be visual. This is done to prevent coaches from stacking boats only with rowers with VI, as VI minimally negatively impacts boat speed compared to physical disabilities. Until recently, all rowers classified with a VI had to wear blackout masks for visual equality to account for differing levels of VI. Currently, blackout masks are no longer required for any rower, but there are eligibility restrictions for certain boats and events if a rower’s VI is not severe enough [4]. At this elite level, Para-rowers, including those with VI, undergo similar high intensity and periodized training to prepare for competition as rowers without disabilities [5].

Within the sport of rowing, coaches and peers can influence the experiences of rowers and can play a significant role in their motivation and enjoyment of the sport [6,7,8,9]. These positive or negative experiences reflect rowers’ perceptions of the supporting and thwarting of their autonomy (actions are in line with interests and values), competence (control over success), and relatedness (sense of belonging) by their coaches and peers. These needs can be influenced by how rowers are selected for a team and boat lineups, as well as how they are provided feedback [8,9,10]. When supported, rowers can have more positively balanced affect, are more persistent, and express more interest in future task engagement; however, when thwarted rowers can express ill-being and disaffection [6,7,8,9].

Similarly, in the parasport and Paralympic domains, athletes’ positive perceptions of coaches’ professionalism (familiarity with the disability types and needs within their sport), collaboration (working with their athletes to facilitate development), and consideration (including the needs of their athletes and reflecting on their own biases) can have significant influence on their sport experiences and participation [11,12,13,14]. In addition to coach empowerment, at the Paralympic level, the notion of belonging to an elite group of athletes as well as the enjoyment associated with participation serve as the main facilitators to participation [14]. However, coach prejudice can have a negative influence on parasport athletes’ experiences and participation [12]. This prejudice can perpetuate socially imposed structural barriers to their athletes’ success, such as coaches being overprotective and not allowing parasport athletes to participate due to fear of them being injured [12,15]. This fear stems from lack of experience seeing athletes with disabilities engaging in typical sports and activities [15]. On the Paralympic level, barriers to participation also include sport-related health complications, deselection, and lack of time and financial support to continue with training [14].

While there is limited research on elite athletes with VI, and even further limited when related to rowing, current research has shown that there are many barriers to participate in physical activity for youth with VI. These barriers range from parental overprotectiveness, to attitudes, lack of knowledge, equipment, and lack of programming [16]. Youth with VI have the right to access the same activities as their same age peers. They deserve the Dignity of Risk meaning that they should have the same options as their peers to get hurt and fail [17]. In order to improve this situation, it has been suggested that youth with VI learn how to self-advocate for themselves in sport and physical activity [18]. The ability to self-advocate has shown to improve the chances of being included in sports and recreation [19]. Advocacy is essential for runners with VI to not only ensure they have a guide to run with, but that they and their guide are welcome to run in races with sighted runners [20]. While the addition of a guide in a sport like running can make it accessible for athletes with VI, rowing is differentiated as a sport, because no guide and minimal modifications are needed [21].

One outlet for athletes with visual impairments is the United States Association of Blind Athletes (USABA) offering opportunities from the regional to international levels of competition. Goalball is the most commonly sport participated in by blind athletes, followed by track and field, alpine skiing, and swimming. While rowing is not one of the more popular USABA sports in which athletes with VI participate [22], the task demands of the sport of rowing allow it to be accessible for individuals with VI [2,21]. Elite rowers with VI are a unique population because they have the potential to be just as competitive outside of Para-rowing as sighted rowers. The combination of being individuals with VI, and elite athletes that are as competitive as their sighted peers, warrants exploration of this unique population’s experiences as well as how this combination influenced those experiences [13,14]. The purpose of this study was to explore the lived experiences of elite rowers with VI in relation to the benefits and challenges of those experiences as well as their peer and coach relationships.

## 2. Materials and Methods

### 2.1. Approach

In order to explore the elite rowers’ experiences with their involvement in rowing, this study adopted an Interpretative Phenomenological Analysis (IPA) approach to guide data collection, analysis, and interpretation [23,24]. The qualitative research approach of IPA examines how participants understand their personal and social worlds, and the meaning their experiences within those worlds hold for them [24]. IPA is considered phenomenological in that it focuses on individuals’ personal perceptions of lived events without any theoretical preconceptions [23]. In addition, IPA focuses on the fact that the research endeavor is a dynamic process in which interpretative activity is necessary for the researcher to understand the participants’ personal world. In this current study, the researchers retrospectively examined the participants’ lived experiences with their journey in becoming elite rowers, while also understanding the significance they encountered in those experiences.

### 2.2. Participants

Participants were recruited based on the following eligibility criteria: (a) having rowed with a VI at the national and/or international level, and (b) were willing to participate in a recorded interview over video conferencing software. Due to the small available sample of participants, elite athletes in this study were defined as having rowed at the national level or higher. Participants were recruited through internet searches or identified through snowball sampling, where initial participants provided contact information of eligible peers following their interview. All participants were contacted through an email request that included the purpose, eligibility criteria, time commitment, and incentive for participation (USD 50.00 gift card) in the study. Interested potential participants were instructed to click a link in the email to read and digitally sign an informed consent outlining the study’s procedures. All of those who did were contacted by the lead researcher and invited to schedule a time for an interview. The study was conducted according to the guidelines of the Declaration of Helsinki and approved by the Institutional Review Board of SUNY Brockport (00002591, 9 October 2020). The pseudonyms Participant A through Participant H are used throughout the paper to protect the participants’ identities.

Eight participants (22–66 years old; *M* = 40.5; *SD* = 16.3), representing three countries, who met the eligibility criteria, provided informed consent, and completed video interviews were included in this study. Seven of the eight participants were female, and one participant was male. Five of the participants’ VI were congenital. Due to the very small population of elite rowers with VI internationally, their cause of VI has been omitted to protect the identities of the participants. Four participants reported having B3 vision (i.e., low vision, visual acuity over 20/600 and/or visual field of 5 to 20 degrees in the best eye and/or a visual field less than 5 degrees), two reported having B2 vision (i.e., travel vision, visual acuity up to 20/600), and two reported having B1 vision (i.e., blind, no light perception in either eye), per the USABA Visual Impairment Classification System [25]. Six of the eight rowers began their rowing in high school or college, with two starting their rowing careers in their 30 s. All participants rowed for their country’s national team on the international level as Para-rowers. Further demographic information and demographics specific to each participant have also been omitted to protect the identities of the participants.

### 2.3. Data Collection

Each participant engaged in an audio- and video-recorded semi-structured interview with two researchers through video conferencing software. The interviews averaged 57 min long. Two researchers were present in each interview to ensure trustworthiness and reduce bias by enabling comparison of reflective notes [26,27]. For continuity, the first author participated in all eight interviews, while the other three authors rotated attendance at the interviews based on their availability. Video conferencing was used to better simulate in-person interviews and allow the researchers a view of participants’ non-verbal behaviors. Each interview began with the researchers restating the purpose of the study and explaining their personal and professional history to exhibit positionality [28]. Interviews then continued with several demographic questions followed by 14 semi-structured interview questions, including open and broadly worded questions regarding the participants’ rowing experiences guided by the IPA research approach. Researchers alternated asking questions, but either researcher could probe at any time, allowing for flexibility and exploration before the next interview question was presented.

The interview questions were created by the lead researchers and focused on participants’ experiences in rowing, including benefits and challenges, teammate and coach relationships, and their self-advocacy. The questions also inquired on the selection process participants underwent at their highest level of rowing, as lineup selection plays a role in Para-rowers experiences and continued participation [14]. Face and content validity were obtained, to ensure the questions would yield answers in line with the purpose of the study, by seeking feedback on the interview questions from six experts in the field: two professors of motor development with expertise in VI, one professor of adapted physical education with expertise in VI, one elite blind athlete who is also an adapted physical education teacher, one professor of exercise science with expertise in rowing, and one professor of sport psychology with expertise in rowing. The feedback from the experts was infused into the interview questions until consensus was reached. The questions served as a guide during interviews to ensure all participants were asked the same fundamental line of questioning but allowed the interview and interviewee some flexibility in what was emphasized [23,29]. Questions were intentionally open-ended to facilitate participants taking the discussion in a direction meaningful to them (see Appendix A).

Reflective notes were taken by both researchers in each interview to serve as a secondary source of data for this study [26]. During each interview, the authors documented notes that included reflections on initial feelings about participants’ responses, responses that seemed meaningful, participants’ non-verbal behaviors, thoughts on potential themes, and the tone of the discussion [26,28,29]. These notes allowed the authors to triangulate the recorded responses and data as well as reflect on any potential biases that may have impacted the interpretation of the interview.

### 2.4. Data Analysis

After the completion of each interview, the audio- and video-recorded interviews were transcribed by research assistants. Participants were sent their interview transcript for member checking to ensure trustworthiness [27]. Seven of the eight participants responded to the member checking follow-up, affirming transcription accuracy. One participant did not respond to the request for member checking. The transcriptions and interview notes were then analyzed independently by two of the researchers involved in the study using a three-step approach in line with IPA to capture and reveal findings in the form of the participants’ embodied experiences [23,24,29]. First, the analysts engaged themselves with the data by reading each transcript and reflective notes several times in order to gain an intimate understanding of the substance of each participant’s transcription. While doing so, the analysts identified responses of interest within the transcripts and reflective notes in the form of descriptive and exploratory notes. Next, the analysts compiled, and condensed transcripts, reflective notes, and descriptive and exploratory notes associated with each participant into constructed themes. In doing so, themes began to reflect both the original responses of the participants as well as interpretations of the analysts. Each participant’s data at this point had undergone analysis independently. The last step of the IPA consisted of identifying prominent and recurring themes across all participants through repeated comparison. Themes identified by each analyst were then discussed collaboratively until agreement was obtained. Then, themes were presented to a third researcher involved in the study who served as a “critical friend” to ensure all themes were accurate and reflective of the collected data [30]. Themes that were agreed upon as fitting the purpose of the study were then compiled and introduced as findings.

## 3. Results

This study examined the lived experiences of elite rowers with VI. The findings of the study demonstrate broad influences and outcomes of those experiences. The analysis identified four major themes: (1) Empowerment Through Rowing, (2) Rowing Through Feel, (3) Changing Perceptions, and (4) Forming Influential Relationships.

### 3.1. Empowerment through Rowing

Throughout their interviews, all eight participants described experiencing a sense of empowerment stemming from their elite rowing careers. Their empowerment presented itself in the form of overcoming fears related to their VI, gaining the confidence and resilience to advocate for themselves, and feeling able to transfer that confidence and resilience beyond rowing. Part of this empowerment was also expressed opportunities that would not have existed without rowing.

Six of the eight participants specifically identified feeling that rowing facilitated opportunities for them, which were presented in diverse ways across the participants. Participants A, D, F, and G described their elite experiences as generating social connections, relationships, and networking. For example, Participant G explained, “It opened a lot of doors for me.” For Participant G, these new doors included speaking engagements, finding apartments, and meeting her husband. Participants D, E, and G also viewed their rowing careers as generating an opportunity to see the world. In her interview, Participant G highlighted, “I traveled the world, so I certainly got to see places that I never would have visited had it not been for my rowing experiences.”

All eight participants highlighted psychological benefits and independence gained from their rowing experiences, even at the non-elite level. Participant A revealed rowing as being instrumental in coping with her vision loss. After losing her vision and being persuaded by a friend to try rowing again, Participant A described her first experience back on the water as the “first time in a couple months where I wasn’t thinking about being blind, and I could just move freely in space with power and not be afraid.” Participant F also attributed rowing to helping her overcome fear of leaving the house related to her VI and was looking for an outlet. She explained, “I wanted to find something that I loved to do so much that I couldn’t stay in the house, like I needed to get out and do it, so rowing did that for me.” Participant F explained that the coxswain steering aspect of rowing helped to abate her fears and apprehensions. She felt that rowing was the only place she could go fast and push, providing her confidence and self-esteem. Participant F illustrated her rowing experiences snowballing and turning her life around, expressing, “It just changed my whole life; I went from being afraid to leave my house to becoming an elite athlete.” With increasing skill, Participant H saw her self-confidence grow along with her comfort in self-advocacy. Participant H, who started as a junior (category for rowers under 19 years of age), believed that if she had started as an adult, it would have been harder to know what or how to advocate for herself. Participant H explained that her experiences advocating for herself in rowing also helped her advocate for other children and athletes with VI throughout her career.

Participant B detailed how her experiences as an elite athlete taught her time management and discipline. She noted, “[Rowing] gives you a lot of discipline. I was pretty much a student through my entire rowing career. I know how to prioritize time.” In striving for improvement, she learned how to set goals and recognized that effort led to improved performance. Participants A, C, D, E, and G described similar realizations and increased mental toughness from their experiences and being able to relate their gained resilience to pursuits outside of rowing. Participant C attributed this gained resilience as a factor in considering training for winter Paralympic sports, such as cross-country skiing.

### 3.2. Rowing through Feel

Throughout the interviews, all eight participants highlighted the accessibility of rowing for individuals with VI, not only as a sport to participate in, but also as an opportunity to be competitive. This is exemplified by Participant C who noted, “[Rowing] gave me something that I could just get in the boat and … it’s not like I have a disadvantage. If anything, I might have even more of an advantage than other people.” Additionally, in describing their early and later experiences with rowing, each participant outlined characteristics of rowing modifications that aided in their performance, as well as aspects of their experiences that were ineffective.

#### 3.2.1. Accessibility of Rowing

Many athletes found rowing more inherently accessible than other sports. Without knowing of her VI or age, college coaches approached Participant B when she was 14 years old for recruitment after witnessing her first time ever on an indoor rowing machine. She recalled:

In middle school, after I was diagnosed, I stopped playing volleyball and basketball because I kept breaking my nose because I couldn’t see the ball. I went to a National Federation for the Blind conference, and they had land rowing machines and a competition. They tried to get people interested and they’re like just go for a minute and see how far you can go. I’ve never rowed, and I did that, and I guess I did really well because I’m pretty tall, so I beat a bunch of Paralympic goalball players, and they’re like wow you must be a rower … and [a] college coach was like when do you graduate?

By the time she was rowing in college, Participant B was considered for, and able to row in, any seat available. However, three of the participants, including Participant B, described their athletic careers not beginning so easily and detailed their disastrous early experiences with ball sports. Growing up, Participant D had played a variety of ball sports, but found her friends excelling at a faster rate. She described getting involved with rowing simply because it did not involve a ball.

Six of the participants revealed a quick transition to rowing with their VI not being a limiting factor. However, Participants G and H outlined that it took them about a year to reach that point, but by their second year, they started to identify gaps in their performance to be related to technique, experience, and effort rather than vision. Similarly, Participant F considered her passion, hard work, and love of the sport to be the ingredients of her success, rather than being selected for international competition solely because she had a VI. She credited this phenomenon to the accessibility of rowing. Half of the participants attributed the accessibility of rowing to the sighted coxswain. Participant F expressed how her trust in her coxswains’ steering and provision of frequent technical feedback and verbal encouragement allowed her to maximize her power.

#### 3.2.2. Strategies and Modifications

Participants B, C, F, and H described rowing in stroke seat (seat in the back of the boat, in front of the coxswain, that sets the rate for the other rowers to follow) to be one of the most accessible seats in the boat, with seven of the eight participants having competed at one point in this position. They explained the accessibility stems from rowing as stroke seat allowing rowers to set the rate, feel the boat, and not have to struggle trying to follow. During her interview, Participant F noted:

When I was having difficulty with timing was when they decided to move me to stroke seat and that made all the difference because I didn’t have to worry about following and that’s how I learned the rhythm of the stroke and everything.

Participant F considered her coaches’ decision to move her to stroke seat as one of the best decisions regarding her performance.

Participants A, E, F, G, and H highlighted that much of rowing is based on feel, regardless of sight. They detailed that the shape of the oar and oarlock allow rowers to feel and hear when their blade is parallel (feathered) or perpendicular (squared) with the water. Participant A revealed that individuals can feel the rate of the boat by feeling the boat move under them, as well as the sound of the rowers in front and behind an individual. However, she detailed a specific drill, “Cut the cake”, as not being accessible, because the point of the drill is to teach following to sighted rowers without auditory or tactile signals. To promote the feel of the boat, Participant H recommended utilizing a fast-hands, slow-body recovery (when the oar is out of the water between strokes) technique. To supplement the feel of the boat on when to end the recovery, Participant G described cutting a tennis ball in half and duct-taping it to her rigger (a metal bar that extends from the side of the boat and serves as the fulcrum of the oar and connects the oar to the boat) to indicate how much reach to get each stroke.

To better understand the feel of the boat, Participant C described her coach having her use sliders (equipment that connects multiple rowing machines together). Participant C explained that she wanted to stroke in the sliders, but her coach wanted her to learn to follow. She attributed this to helping her become a better and more versatile rower, and she would continue to collaborate with her coach on where in the boat to sit to be accessible but also push her threshold. Conversely, Participant G detailed that sitting in bow (front of the boat, furthest from stroke) was less accessible, because there is no momentum behind the rower to feel to help with timing. She described wanting her coach to put her in three-seat (two seats behind bow), rather than bow, so she would have rowers on both sides and would progress faster.

Although each participant detailed different strategies that assisted with learning, all the participants detailed that no additional equipment was necessary for rowing and racing with a VI. Though blackout masks are no longer required for athletes to race with a VI, Participants B, F, and H had mixed opinions about the modification. Participant B found the blackout masks to be effectively used by her junior coach to teach her sighted peers to feel the boat and described her coaches utilizing eyes-closed rowing drills as well. Participant H also supported rowing with eyes closed or blackout masks, and highlighted:

When I went back to normal rowing afterwards, I actually missed the blindfold because I felt I was actually getting more from the boat with a blindfold on, and I could understand what the boat was doing and find the faults in the boat better with the blindfold. As soon as I get used to normal rowing again, I really missed it. It was like there is a very, very strange sensation. You’re feeling like your vision almost impeded you when you got back to normal rowing that it was like, I’m not really feeling that anymore, you know, because your visual sense took over.

Conversely, Participant F discussed that the blackout mask removed her light perception and made it difficult to maintain balance and body awareness as she fatigued; she relied on that tiny amount of vision she had. She described having a mini panic attack putting on the blackout masks as she felt completely disconnected from her senses.

The participants revealed that rowing machines on land are also accessible, because their monitors provide audio feedback. Participants B and F reflected that one of the more effective coaching techniques they experienced was their coaches teaching them to row using tactile feedback with the coaches’ hands on the rowers’ hips and backs while they were on the rowing machine. They explained that this is the same technique used for sighted rowers. As Participant B became more adept on the rowing machine, a partner rowing next to her would provide verbal feedback if she had an issue with her technique.

Similar to the rowing machine, Participants D, F, G, and H found it helpful to receive verbal and tactile feedback off the water, after practice, by utilizing equipment, such as a dock box (i.e., static box attached to a dock that allows rowers to put their oar in the water and simulate rowing) or rowing tank (static boat in a room with channels of water to simulate the feel of an oar in the water), to manipulate their stroke. Additionally, they expressed frustration at their coaches who would demonstrate from the motorboat and found it more effective for their coaches to physically guide them with their motorboat alongside their boat, a practice already used for sighted rowers.

### 3.3. Changing Perceptions

In recalling their rowing experiences, seven of the participants highlighted experiencing a mixture of stigma and acceptance of their disability, with the former coming from those who did not know them personally and the latter from those with experiences with individuals with VI. As an example, Participant A recalled, “Once they start rowing with me then people really embrace the idea of a blind partner and that it’s not a big deal.” Though the example provided is of eventual acceptance, the participants’ interviews outlined examples of ableism derived from the stigma of VI as well as their efforts to formally and informally educate their coaches and peers towards that acceptance of their disability.

#### 3.3.1. Ableism

Spotlighting early ableism in her rowing career, Participant A described the first time she tried to row for a club program after losing her sight; the boathouse manager considered it a huge liability. Participant A expressed:

I wanted to go out and the boathouse manager was like freaked and wasn’t so sure that she wanted me to be rowing even though she knew me, and she knew my rowing skills. She thought this was a huge liability, so she stood on the dock and watched and then broke up laughing about 10 strokes and said, ‘I don’t know what I was afraid of.’

Participant A also detailed that in the time period she rowed, most boathouses would not have even given her that chance or trust. Highlighting this stigma, Participant B detailed, in her first attempt to participate in rowing, being rejected from joining a co-ed club for being blind. Even with years of success and experience in rowing with a VI, Participant H, while training to row a single in a popular regatta, received information that the regatta organizer made derogatory remarks towards her VI. As a result, she opted to not compete.

Participant A described experiencing stigma on her club teams that she would not do her share of the work, such as helping to carry the boat from the boathouse down to the dock and pulling her own weight on the water. To overcome this, Participant A advocated to carry the boat down with her peers. Participant F recalled having a similar experience with her coaches, in which they would not originally let her carry the boat down. She also expressed feeling self-conscious when she started rowing about being singled out because she required special accommodations. Participant C found communication with her coaches about slowing down the logistical part of practice helped her. She elaborated that there is normally a time limit in practice to get a boat from the boathouse into the water to mimic race procedures. Participant C expressed that this negatively incentivized her peers to do the work for her, until her coaches intervened by giving more time to carry the boat down.

Four of the participants expressed frustration at a lot of people confusing the Paralympics with the Special Olympics or not understanding the significance of their achievements. Participant H recalled that people at her club would make comments about how Para-rowers, at the time, would only row 1000 m. She provided the example of, “You’re only Paralympic you know, you’re not the real thing”. She described the changing of Para-rowing to 2000 m as being really good for the perception of rowers with disabilities. Participant C similarly described frustration with people’s lack of knowledge that Para-rowing even existed. Participant F highlighted that even after medaling in a world championship, when she returned to her club, the club’s coaches and administrators treated her like a novice but still promoted her for publicity. Participant A explained that during her time on the national team for Para-rowing, she experienced stigma from non-Para national rowers. According to her, it appeared the Para-rowers were not accepted as athletes who deserved to be there by the non-Para national rowers. Participant A highlighted that their attitudes did change after her boat won all the rowing-related medals at that year’s worlds.

#### 3.3.2. Education

Half of the participants expressed perceptions of stigma and ignorance from their coaches and peers on their capabilities while in a boat, but three of those participants felt with communication and time those perceptions were replaced with understanding. In college, Participant G felt like her college peers considered her an anchor when she was in the boat. She recalled they would say, “Oh great, she’s in the bow”. However, Participant G felt that these perceptions dissipated during her second year at which point she considered her performance not limited by her VI. Participant H described a similar feeling of being an anchor when rowing with people who did not know her; however, she did not feel this way when rowing with friends who knew her VI was not a disadvantage. Participant A’s national-level coaches were apprehensive about pushing or correcting her and her peers when they first began. It took them some time to realize that while Participant A’s boat had athletes with disabilities, they were athletes like everyone else. Participant A emphasized to her coaches to yell at her if they felt that it would help correct her, but if they were not descriptive, she may not understand their corrective feedback. Participant B described that her college coach refused to put her in stroke seat because she could not see, despite her experience stroking on the junior level. She even presented to her coach quantitative data on the effects of her stroking upon the overall boat speed, but even the data did not change his mind. She expressed that while the coach had bias about her ability to stroke with a VI, he was more than willing to have Participant B engage in publicity interviews after practice because she was a high-level rower with a VI.

Three of the participants described a lack of coach awareness and training regarding VI outside of the Para-rowing level. Participant F and H highlighted that awareness did not exist at their local clubs. They expressed needing education for club coaches to be aware of the need to move boat parts and other obstacles left on the floor in the path of rowers with VI. They revealed feeling frustrated when coaching changes occurred and they had to start over and re-educate them. They felt lucky to have made it to the national team quickly and having that support. Participant F described her Para-coaches as advocates but the team managers as lacking awareness. She mentioned that the national team coaches of rowers without disabilities could also benefit from increased awareness to see the national teams as one team rather than us and them. Similarly, Participant G detailed that while her college coach was supportive and wanted to teach in accessible ways, the coach lacked the expertise, patience, and creativity to get Participant G to the competitive level she was striving for. Conversely, Participant G felt her Para-coaches knew how to best support an athlete with VI.

Participants C and H recalled that many of their peers without VI, even on the Paralympic level, would forget they were visually impaired, because of the lack of visibility of their impairments, such as not having a white cane or a dog. At times, their coaches would forget about their VI and not be descriptive enough, which led to miscommunication. Participant C described slowly opening up to her college peers about her VI as it progressed over the years. By her junior year in college, she started to use a cane and had to explain to teammates what she could see. As her comfort level increased with discussing her VI, she began advocating for herself more and found her college team to be very accepting. With every incoming class of freshman rowers, Participant C would have a chat explaining her VI and needs.

### 3.4. Forming Influential Relationships

All participants highlighted that their relationships with their family, peers, and coaches played a significant role in their rowing experiences at all levels of the sport. As an example, Participant A highlighted:

I think there was a stronger bond even with our national team because of the fact that we all shared the fact that we had a disability. There was just sort of a camaraderie about being able to move the boat together and race together and deal with what we were dealing with and kind of appreciate it even though our disabilities may not be the same.

While family members facilitated early involvement in rowing, the rowers’ relationships with peers and coaches determined a sense of camaraderie and belonging on their respective teams.

#### 3.4.1. Peers

In addition to initial and continued support from family, each participant identified their teammates as having a significant role in their experiences. A draw to the sport for all the participants was the camaraderie of practicing and racing together, and the relationships formed thereafter, describing their peers as family. Participants C and E detailed that it stems from going through a lot of pain and hardship together and sharing those experiences. Participant C considered that level of connection something exclusive to rowing. Three of the participants detailed a heavy support from a single peer on the national level. Participant B explained that her teammate helped her train and taught her how to set and achieve goals. Participant C also described her national team training partner as like a brother to her. While Participant D is a B2 (i.e., travel vision, visual acuity up to 20/600), she described having a B1 teammate on the national team who made her realize that vision cannot hold her back from anything, only she can. It was illuminating for her that her peer never once said he could not do something because of his lack of vision.

Participants F and H described having a good relationship with their Para-rowing peers because everybody had a disability, and the environment was inclusive. An aspect of the sport that grabbed Participant F was she felt she had a lot in common with her teammates because they were high functioning, had started a family, and had careers. In previous non-rowing adaptive groups, she had joined, she felt more like she was helping than participating. It was motivating for her to meet people at a national competition who were pursuing their careers, such as law. She felt she could relate to them and appreciated they could all laugh together about their disabilities. Participant F viewed that laughter as a coping strategy. She described that her teammates looked out for each other all the time, and when one was stressed, the others served as a guide to help them get through it. Similarly, Participant H attributed a sense of belonging to her success. She loved the attitude of the Para events and considered it a perfect world for a person with a disability.

Not all peer interactions were positive. Participant B identified some struggles with her peers when training on the national team. Aside from her supportive peer in the coxed four, her other teammates would dismiss her for her lack of experience, not listening to her or taking her feedback. If she were stroking, they would pin any issues on her. She highlighted that her coxed four had a variety of motivations and goals and did not row with a single boat goal. She did not feel connected to her team and attributed that feeling to hurting her performance. Participant B expressed that these poor relationships on the national team led to her leaving the sport.

Participant C also reported some difficulties trying to juggle college rowing and the national team. As she progressed on the national level, she started to feel more connection with her Para-rowing team than her college team. She described having trouble transitioning back to college after training with her national team Para-rowing peers. In her interview, she expressed, “I just realized that I felt so much more like my authentic self with the Para-athletes because we all have disabilities, and we all know what it’s like to have people judge us.” Participant C described while having a great relationship with her college teammates, she did not feel like they had that kind of empathy. She “felt like [she] was away from [her] family”, meaning, her Para-rowing peers were her family as opposed to her college team. Compared to her Para-rowing experience, Participant C acknowledged receiving different feedback from her college coaches and feeling like it was a different environment. She expressed having trouble being present when rowing at college, because her training goals were directed toward the national team and not the college team. She did not care for the conference and regional championships for her school and only viewed her college benchmark test improvements as performance indicators to be accepted to go to national camp. Conversely, Participant G reported not having as strong of relationships with her national team peers as her college peers because she did not see them as often. Participant G described wanting to keep it more competitive on the national team level by not getting too close to her peers.

#### 3.4.2. Coaches

Seven of the eight participants outlined how their relationships with their coaches impacted their perceptions of their experiences. At the college level, Participant D felt that her coach had effective communication and connection and served as a role model for her own coaching. She recalled:

You always hope to have a great relationship with your coach, and I feel like I’ve had that for the majority like really great communication and great coaching and fantastic role models. You always have a few coaches who aren’t in it for the right reasons or feel a little bit against you and whatnot, but I’d say I had a really great experience, especially with my college coach. I mean we’re still close, you know; I can count on her for anything.

Participant D expressed appreciation that her coach gave her honest, performance-based feedback, and along with Participants C and G, she recalled that her college coaches did a wonderful job of making her feel accepted. Similarly, Participant B highlighted that her junior coach was very influential to her experience as he promoted a shared purpose and identity on the team.

On the national team level, Participants C, E, and F detailed enjoying their relationships with their coaches. They recalled that their coaches always tried to solve problems and consider how they could make them faster. Participants G and H reported having a positive relationship with one of their many national team double coaches. As a result, they did not feel the same pressure and high expectations as other rowers. Participant H explained that her favorite national team coach “just knew” when it came to working with her. He asked questions when he did not understand, involved her in feedback, and was not afraid of working with her impairment. She recalled respecting him because he treated her like a sighted athlete and had prior success with one of her B1 peers.

While three participants described aspects of negative connections with their coaches, Participant B expressed that her relationship with her most recent coaches pushed her away from the sport. Participant B reported a diminished rapport with her college coach and explained that he had lost legitimacy and respect from her and her teammates because he did not have high level rowing experience. She detailed that he was very transactional and manipulative with scholarships and team positions if he did not like a rower or if a rower got sick or injured. Participant B expressed that her college coach not listening to her feedback or providing transparency, objectivity, and consistency in his lineup selection and feedback left her unsure if his decisions were based on her vision or her performance. Participant B described her college coach creating castes on the team: those who were fast tracked to the Olympics, those who the coach had a gut feeling about, such as his daughter and international rowers, and everyone else. However, Participant B was not getting what she needed from the national team either. She felt her national team coach did not have a direction or structure. Participant B expressed a lack of ability to progress and control her performance on the national team, cutting her motivation. She recalled a lack of support after an injury and feeling her opportunities were ruined. She expressed these experiences and relationships playing a role in her departure from the sport.

## 4. Discussion

The purpose of this study was to explore the lived experiences of elite rowers with VI in relation to the benefits and challenges of those experiences as well as their peer and coach relationships. As a result, four major themes were identified that demonstrated the influences and outcomes of those experiences: (1) Empowerment Through Rowing, (2) Rowing Through Feel, (3) Changing Perceptions, and (4) Forming Influential Relationships. As there is limited research on this unique population, the findings of this study can begin to discern the barriers and facilitators of those experiences and their impact on rowers with VI’s motivations, performances, and enjoyment of the sport.

Aligning with previous research on Paralympic athletes, the participants’ described perceptions of their coaches’ and peers’ collaboration, consideration, and familiarity with their disability and needs positively influenced their experiences, while much of the rowers’ negative experiences stemmed from perceived coach and peer prejudice [12,13,14]. These described positive and negative experiences also reflect the rowers’ perceptions of the supporting and thwarting of their autonomy, competence, and relatedness by their coaches and peers throughout their rowing career, and how those perceptions impacted the rowers’ autonomous motivation and enjoyment of the sport [6,7,8,9]. The coaches who best supported the participants’ acceptance and sense of belonging (relatedness) were also the ones to best facilitate the participants feeling in control of their success (autonomy) [8,9,13]. This acceptance came through collaborations and problem solving on how best to accommodate for their performance and success [11,12,13]. However, Participant B’s college coach’s lineup decisions served as a barrier to her feeling competent and in control of her success because she was put in a more disadvantageous seat for her VI, despite data showing that the boat was fastest when she was in stroke [8,9,11,13,14]. Participant B’s reflection of how her national team’s loose structure and lack of support after injury also aligns with previous research on the thwarting of autonomy, competence and relatedness and explains why she then experienced decreased autonomous motivation and enjoyment of rowing, leading to her departure from the sport [6,7,8,9,13,14].

The participants carrying the boat down to the dock played a role in feeling a sense of belonging and acceptance on their teams [8,9,21]. Being prevented by a coach from doing all the tasks required and expected of a rower due to their VI has the potential to make a rower feel removed from their team or like they have no room for improvement in those tasks because there is little they can do about their disability, thus thwarting relatedness and competence [8,9,11,12]. This finding is remarkably similar to the overprotection of parents, teachers, and coaches that has been found in the literature for years (see [16]). The act of protecting the participant from engaging in the sport, physical activity, or fitness due to fear of injury or failure continues to put limits on people with disabilities and in this case continues even in very accessible sports such as rowing [15]. The participants who were turned away by clubs or coaches were made to feel like they were not accepted by the rowing community before they even started [11,12,14]. Though not the case of any of the participants, an early stigma-based barrier to participation or prejudiced comment could have ended their rowing career before it began, such as when Participant H did not row her single in a regatta because of the organizer’s attitudes. This aligns with the issue of Dignity of Risk by taking away the opportunity to participate just because others think they may get hurt or not be good enough [17,20,22]. The notion that a person with a disability, in this case a VI, cannot have the same choice to participate and get hurt is one that advocates are trying to minimize in order to ensure every individual has equitable opportunities to engage in the same activities.

The rowers’ discussion of their peers embracing the idea of a boatmate with VI illustrates Participant C’s described increased connection inherent to rowing and its ability to facilitate relationships and belonging (relatedness) regardless of VI [8,9,14]. These connections were further enhanced on the national team, because of the camaraderie and empathy of all having a disability and going through the experience of moving the boat together [14]. Participant C’s feelings of authenticity stemming from those connections explains her described lack of motivation and being present on the collegiate level due to perceptions of the relative levels of autonomy and relatedness between those competitive environments [14].

The accessibility of rowing allows for a greater sense of belonging (relatedness) for rowers with VI because of their potential for success [8,13,14]. Participants A, G, and H felt accepted by their college and club peers once the peers recognized that there was no disadvantage to rowing with a VI. The shift of Para-rowing to 2000-meter races served to also help the acceptance of the participants as “the real thing” [3]. However, Participants B’s and F’s experience of being used for their international accolades while at the same time feeling excluded or shunned by their club and college, respectively, exemplifies the dichotomy between rowing with VI for the national team and rowing for a junior, college, or club team [14].

Participant C’s description of being involved in a sport where she not only does not have a disadvantage, but potentially an advantage speaks to her feelings of competence in rowing [13,14]. For this same reason, ball sports did not provide Participant D with the feelings of competence that Participant C described with rowing, leading to Participant D’s transition to the sport. The participants’ quick transitions and early feelings of awareness of control in their performance can be attributed to the various highlighted aspects of rowing that promotes its accessibility [8,13,14].

The steering and verbal communication role of a coxswain removes the most visual factor in rowing, but also includes a feedback role that is inherently accessible for rowers with VI [2,21]. Fulfilling part of a coxswain’s role on land, the ability for rowing machines to provide performance feedback, such as speed and stroke rate, adds to the rowers having more control over their performance and improvement in training [10,21]. Rowing as stroke seat, allows rowers to set the rate, feel the boat (“boat feel”), and not have to struggle trying to follow [21]. As such, the seating and position decisions Participants C’s, F’s, and G’s coaches made played a role in their athletes’ accessibility and potential to progress (competence) [8,9,14,21]. The described coach-guided verbal instruction and tactile modeling on the rowing machine and water also allowed the rowers to feel competent as the feedback was accessible to them [10,20,21]. This accessible verbal and tactile feedback were facilitative to their experiences and improvement in the sport, whereas inaccessible visual feedback only led to frustration and confusion [10,11].

Each participant’s highlight of how their experiences provided psychological and social benefits illustrates an exerted control over their lives, careers, and successes, allowing the rowers to regulate their life in line with their passion and values (autonomy) [11,14,20,21]. As an example, Participant H chose to reciprocate the autonomy she gained from learning to advocate for herself by paying it forward and advocating for children and athletes with VI in her career [14]. Similarly, Participant C’s consideration of training for a different sport, as well as Participant F’s overcoming of her fears to leave her house, illustrates how the resilience and autonomy they gained from their rowing experiences has lowered self-imposed barriers for them as athletes with VI [14,20].

### Practical Implications, Limitations, and Future Research

There are several practical implications for coaches to positively influence the experiences of all levels of rowers with VI. Coaches of rowers with VI have the potential to facilitate accessibility for those rowers through providing effective instruction and feedback, promoting “boat feel”, and fostering an inclusive environment [21]. Coaches can promote “boat feel” through teaching a quick-arm style of rowing and placing rowers with VI in stroke seat [21]. Coaches of rowers with VI should provide auditory and tactile feedback and should consider pairing the two together on land and water to increase their efficacy [21]. All coaches should continually reflect on their disability biases and expectations. Lastly, it is important for coaches to recognize that not all errors may be related to VI and coaching decisions should be informed by performance-related data rather than bias [21].

Along the same lines, it is clear in this study that the ability to self-advocate for the rowers’ needs helped them get what they wanted. Self-advocacy is necessary for every individual with a VI to ensure they are being provided access to their needs [18,20]. Seat position, tactile instruction, and verbal feedback of visual information were all extremely helpful to these elite rowers. Ensuring young and new rowers learn their choices in the sport of rowing and how to ask for it is tantamount to an overall successful experience for everyone. While some of these implications for coaches are rowing-specific, many are transferrable to coaching athletes with VI in other sports [20,22].

The limited population size of eligible participants with VI who rowed on the national level or international level served as a limitation to this study. Additionally, limited research on sighted Para-rowers or other non-rowing elite athletes with VI creates a gap in the literature to compare the participants’ experiences. As this was a qualitative study, there is a limitation on the generalization of its findings. Future research should explore the experiences of other populations to provide context to the findings of this study, as well as further understanding and awareness of Para-rowing and athletes with VI. Future research should also investigate how best to provide education to coaches on the needs of athletes with VI.

## 5. Conclusions

Rowing is an accessible sport that can be enjoyed by athletes with and without disabilities. The sport experiences of rowers with VI, on all levels, are heavily influenced by their interactions and relationships with their teammates and coaches. This study illuminates the barriers faced and the variables that facilitate active involvement on the team for rowers with VI. These considerations and strategies can help drive the planning and implementation for the coaches and administrators of these rowers to best support their autonomy, competence, and relatedness, leading to improvements in their motivation, performance, and enjoyment of the sport. With some education for coaches and administrators as well as careful considerations related to accommodations, rowers with VI can gain extensive physical, psychological, and social benefits from participating at the junior, college, club, national, and Paralympic stages.

## Data Availability

The data presented in this study are not available for privacy and ethical reasons.

## Appendix A

Interview Questions

1. How did you get involved in rowing?
2. Tell me about your experiences when you first got involved.
3. How did these experiences change as you progressed through your rowing career?
4. What do you find to be the benefits of your rowing experiences?
5. How have you needed to self-advocate, if any, during your rowing career?
6. What do you attribute as helpful to your success in your collegiate rowing and onward?
7. What do you attribute as a challenge to your success in your collegiate rowing and onward?
8. Tell me more about your relationships and interactions with your teammates and boatmates during your collegiate rowing and onward.
9. Tell me more about your relationship and interactions with your coaches during your collegiate rowing and onward.
10. How did your coaches attempt to motivate you?
11. How effective were each of their methods to motivate you?
12. Tell me about the lineup selection processes you underwent in collegiate rowing and onward?
13. How would you change anything, if any, about those selection processes?
14. Tell me about other influential people in your life that contributed to your rowing experiences and career.
